# Canagliflozin on top of dual renin-angiotensin system blockade in a woman with partial acquired lipodystrophy, type 2 diabetes and severely proteinuric chronic kidney disease: a case report

**DOI:** 10.3389/fendo.2023.1172468

**Published:** 2023-05-19

**Authors:** Edoardo Biancalana, Giovanni Ceccarini, Silvia Magno, Valerio Ortenzi, Domenico Giannese, Ferruccio Santini, Anna Solini

**Affiliations:** ^1^ Department of Clinical and Experimental Medicine University of Pisa, Pisa, Italy; ^2^ Obesity and Lipodistrophy Center, University Hospital of Pisa, Pisa, Italy; ^3^ Section of Pathology, University Hospital of Pisa, Pisa, Italy; ^4^ Nephrology Unit, University Hospital of Pisa, Pisa, Italy; ^5^ Department of Surgical, Medical, Molecular and Critical Area Pathology, University of Pisa, Pisa, Italy

**Keywords:** dual renin-angiotensin system blockade, proteinuria, canagliflozin, lipodystrophy, metreleptin, case report

## Abstract

Sodium glucose cotransporter 2 inhibitors have proven strong efficacy in reducing end-stage renal disease in patients with type 2 diabetes. We are presenting here the case of a 40-year-old woman with acquired partial lipodystrophy, type 2 diabetes and essential hypertension complicated by chronic kidney disease and proteinuria in the nephrotic range. She first came to our attention in 2012; estimated glomerular filtration rate (eGFR) was 41.5 ml/min/1.73 m^2^ and total proteinuria was 375 mg/24h; she was treated with dual renin angiotensin system blocking. Proteinuria significantly increased during the following years, reaching a nephrotic range (>5 g/day). A kidney biopsy revealed a tubule-interstitial involvement compatible with type 2 diabetes. Leptin replacement therapy, started in 2018, improved glycaemic control and lipid profile, also determining a reduction in insulin total daily dose. In 2019, after the publication of the CREDENCE study, canagliflozin was started on top of losartan and ramipril. After an initial, expected eGFR drop, kidney function stabilized, and albuminuria significantly reduced (from 4120 to 984 mg/24h), while serum potassium showed only minimal increase. At last follow-up (2022) total proteinuria was still reducing (510 mg/24h), while kidney function was substantially unchanged (eGFR 40 ml/min/1.73 m^2^). This case report suggests that, despite not recommended in international guidelines, the use of SGLT2i in combination with dual renin angiotensin system blockade should be considered in specific conditions and under close clinical monitoring.

## 1 Introduction

Sodium glucose cotransporter 2 inhibitors (SGLT2i) are now recognized as the first therapeutic choice for the treatment of chronic kidney disease (CKD) of both diabetic and not diabetic aetiology. Several clinical trials have documented as SGLT2i are able to slow eGFR decline and reduce albuminuria (1). The CREDENCE trial, published in 2019, enrolled severely albuminuric participants with baseline eGFR between 30 and < 90 ml/min/1.73 m^2^ (2). To date, other studies have been conducted in patients with even lower eGFR: the DAPA-CKD recruited participants with T2D and eGFR ≥25 ml/min/1.73 m^2^ and the EMPA-KIDNEY enrolled subjects whose eGFR was ≥20 ml/min/1.73 m^2^, confirming the efficacy and safety of SGLT2i in such advanced stages of kidney disease (3, 4). These drugs are now proposed as a first line option in all the international guidelines (5).

Lipodystrophic syndromes are a heterogeneous group of rare acquired or congenital disorders characterised by either generalized (GLD) or partial (PLD) lack of subcutaneous adipose tissue (SAT) (6, 7). These disorders may be associated with different forms of metabolic impairment caused by fat accumulation in muscle and liver, with consequent insulin resistance, diabetes, hypertriglyceridemia, and hepatic steatosis. Acquired partial lipodystrophy (APL), is a very rare type of lipodystrophy, including different phenotypes, usually sharing the association with autoimmune disorders. In APL, a reaction against white adipose tissue antigens is postulated (8, 9). Some APL have been known since decades and new forms have been recently described but modest advancement on the characterization of pathogenic mechanisms has been made. When present, renal involvement can lead to kidney failure and proteinuria, significantly impacting clinical prognosis (7). Treatment of such level of kidney disease in a so complex clinical scenario can be very challenging. Here, we present a clinical case of a patient with APL and type 2 diabetes (T2D) in which the addition of canagliflozin on pre-existing therapy with both angiotensin receptor blockers (ARB) and angiotensin converting enzyme inhibitors (ACEi) markedly reduced proteinuria and slowed eGFR decline.

## 2 Case presentation

A 40-year-old woman came to our attention in 2012 with new-onset T2D and poor metabolic control (HbA1c 82 mmol/mol). She had a medical history of arterial hypertension and, 7 years before, a pregnancy complicated by gravidic gestosis and HELLP syndrome. She also had hypercholesterolemia and severe hypertriglyceridemia requiring pharmacologic treatment. After delivery, blood pressure (BP) progressively increased and renal function started to decline, with rapid onset and persistence of moderate proteinuria. At the first visit, eGFR was 41.5 ml/min/1.73 m^2^ and proteinuria was 375 mg/24h; the microscopic urinary sediment also showed 10 red blood cells, and 31 white cells/high power field and hyaline casts. At physical examination, her body mass index (BMI) was 19.8 kg/m^2^ and BP 126/80 mmHg. Chest and heart inspection, palpation and auscultation revealed no pathologic findings. Peripheral pulses were valid.

The routine analysis showed mild anaemia (Hb 10 g/dl, MCV 87 fL), likely due to folate deficiency (4.3 ng/mL, normal range 4.6-18.7), normal liver enzymes and electrolytes balance. She was treated with losartan 50 mg + ramipril 10 mg, metformin 500 mg BID, glimepiride 2 mg, fenofibrate 145 mg, atorvastatin 40 mg and PUFA. Such RAAS double blockade had been started years before for the recurrency of a relevant proteinuria (up to 3 g/24h) documented several times after the pregnancy. Measures of BUN, creatinine and electrolytes had been performed every six months. No side effects were reported. Blood pressure control was adequate and serum potassium was 5.14 mEq/l, allowing us to confirm a reduced dose of losartan as add-on to ramipril.

The anti-hyperglycaemic treatment was initially intensified by introducing insulin glargine at bedtime, repaglinide and sitagliptin, while glimepiride was withdrawn. A nutritional counselling recommended to limit protein and salt intake according to international guidelines for management of CKD.

In 2013, her glucose control further deteriorated (HbA1c 101 mmol/mol), and the patient was admitted in hospital, undergoing a therapeutical intensification to a basal-bolus insulin regimen plus DPP4-inhibitor (linagliptin 5 mg). eGFR (35.0 ml/min/1.73 m^2^), and proteinuria (798 mg/24h) worsened, with a normal urine sediment. No other alterations emerged at routine analysis, including complete blood count (CBC) and haemoglobin level (Hb 11.5 g/dl, MCV 89 fL). An abdominal ultrasound reported hepatic steatosis, kidneys of longitudinal length (LL) of 101 and 92 mm with hyperechoic cortical parenchyma and renal sinus cysts, aorta with atheromatic plaques. The doppler ultrasonography of renal arteries (RA) showed absence of stenosis, with renal/aorta ratio (RAR) within normal limits and normal renal resistive index (RRI 0.60 and 0.64). She was advised to continue dual RAS blockade with stabilization of eGFR and albuminuria until 2014, when, in face of an eGFR of 45 ml/min/1.73 m^2^, an albumin to creatinine ratio (ACR) of 91 mg/g, mean BP values of 135/80 mmHg and K^+^ 5.2 mEq/L, we tried to withdraw losartan.

In 2015, while eGFR slightly improved (eGFR 54 ml/min/1.73 m^2^), ACR raised up to 358 mg/g, inducing us to reintroduce losartan. Moreover, her BMI was 20.2 kg/m^2^, and she showed a peculiar pattern of subcutaneous adipose tissue distribution characterised by extreme reduction of the adipose tissue in the limbs while it was preserved in the face, confirmed by plicometry and Dual Energy X-ray Analysis, which allowed us to diagnose partial lipodystrophy. To better define the subtype of lipodystrophy, screening for PPARG and LMNA genes pathogenic mutations, responsible of most frequent congenital forms of partial lipodystrophy, was performed and no mutations were found. A complete hormonal profile was then performed, revealing low-normal leptin levels (7.5 ng/ml) without abnormalities in the hypothalamus-hypophysis-axis, thyroid, ovarian and adrenal gland function (**Supplementary Table 1**, panel A). The possible association with autoimmune diseases was also explored; C3 and C4 levels were found to be normal while a seropositivity for ANA and gastric anti-parietal cell antibodies emerged, despite the absence of anaemia (Hb 12.1 g/dl) and iron deficiency (**Supplementary Table 1**, panel B). Abdominal ultrasound was repeated, reporting no significant changes (kidney’s LL of 95 and 93 mm, hyperechoic parenchyma with thickness in the normal range, right renal cyst of 2 cm).

**Figure 1:**
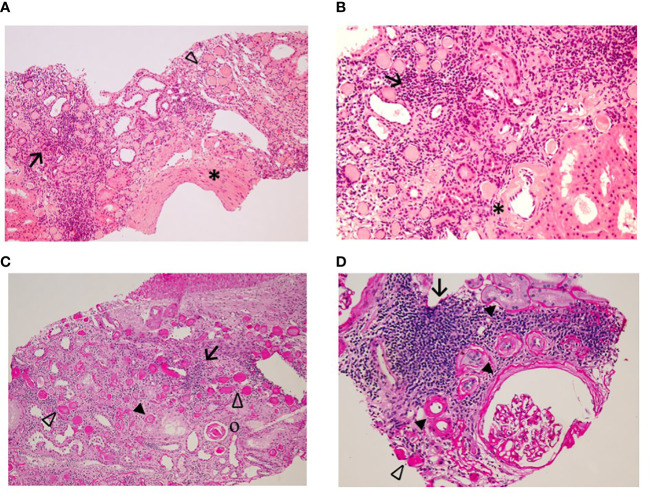
Kidney bioptic specimen showing diffuse lymphomonocytic inflammatory infiltrates (↑) associated with intertistial fibrosis, widespread findings of tubular atrophy (▲) and vascular wall thickening (*) [H&E staining, top images, 100x magnification left **(A)** and 200x magnification right **(B)**]; PAS staining [bottom images, 100x magnification left **(C)** and 200x magnification right **(D)**] furtherly illustrating glomerular sclerosis (o), glomerular capsule and basement membranes thickening (*), plus a number of intratubular hyaline casts (⊳) deeper into the medullary part of nephrons (the so-called tubular «thyroidisation» phenomenon).

In 2016, ACR further increased to 1334 mg/g and eGFR declined to 43 ml/min/1.73 m^2^; K^+^ was 4.85 mEq/L. Losartan was withdrawn again but, at the following control, in 2017, albuminuria was extremely high, reaching a nephrotic range (6000 mg/g) while kidney function was stable (eGFR 50 ml/min/1.73 m^2^ and K^+^ 4.28 mEq/L). The doppler ultrasound revealed a proximal moderate stenosis of the right RA (peak systolic velocity (PSV) of 350 cm/s, end diastolic velocity of 107 cm/s and RAR of 4.0) without signs of renal hypoperfusion (RRI of 0.64-0.69 and 0.57-0.64). Both kidneys presented normal LL (100 mm and 98 mm) and normal parenchymal thickness (16 mm bilateral) with loss of corticomedullary differentiation. Kidney biopsy was then indicated to distinguish between a diabetic kidney disease (DKD), a kidney involvement secondary to lipodystrophy or a primary glomerular disease, like membranoproliferative glomerulonephritis. At microscopic analysis, a tubular atrophy emerged with interstitial fibrosis, lymphomonocytes infiltrates and vascular hyalinosis, all suggestive of DKD (**Figure 1**). In face of such serious proteinuria, double renin angiotensin system (RAS) blockade was reintroduced.

In 2018, the metabolic profile was significantly deteriorated (triglycerides 518 mg/dl and HbA1c 63 mmol/mol). and the decision to start leptin replacement therapy (LRT) under a compassionate use program was taken, with a brilliant response (triglycerides 113 mg/dl and HbA1c 56 mmol/mol after six months). Treatment was well tolerated and consented to rapidly halve insulin total daily dose (from 2 to 0.9 UI/kg in just one year).

In 2019, the results of the CREDENCE trial were published, demonstrating relevant nephroprotection of canagliflozin in T2D patients with advanced CKD and macroalbuminuria. Given that the patient’s eGFR and electrolyte balance had been quite stable during the last 4 years, we decided to introduce canagliflozin 100 mg on top of losartan ad ramipril, strictly monitoring kidney function. The patient was completely involved in this choice: we shared the decision with the medical team and explained to her the clinical rationale and the expected benefits. Renal ultrasound and doppler examination were repeated before starting treatment. The stenosis of right RA was unchanged (PSV 343 cm/s, RAR 3.0) while both kidneys showed slight LL reduction (85 mm and 86 mm), parenchymal thickness still in the normal range (14 mm bilaterally), and no sign of hypoperfusion (RRI 0.63-0.65 and 0.59-0.68).

After an initial, expected, drop (-11 ml/min/1.73 m^2^ after 6 months of treatment, from 38 to 27 ml/min/1.73 m^2^), eGFR gradually improved: after one year it was 32 ml/min/1.73 m^2^, and then further improved to 38 ml/min/1.73 m^2^ later on (**Table 1**). Only a slight transient increase in serum potassium occurred. Moreover, proteinuria rapidly decreased (by 61% in one month, from 4120 mg/24h to 1600 mg/24h): it was 760 mg/24 after 6 months (2020) and 940 mg/24 in 2021 (**Table 1**). The patient was periodically checked with clinical examination and routine analysis exams which did not show significant alterations including CBC (Hb 11.7 g/dl and MCV 91.1 fL in 2019, Hb 11.1 g/dL in 2020, Hb 11.9 g/dl in 2021).

**Table 1:** Clinical parameters over the whole observation period.

	2012	2013	2014 stop Losartan	2015 restart Losartan	2016 stop Losartan	2017 restart Losartan	2018 start LRT	2019 start Canagliflozin	2020	2021 stop Losartan	2022
**Creatinine** (mg/dl)	1.55	1.77	1.37	1.16	1.41	1.23	1.63	1.59	1.82	1.65	1.55
**eGFR** (ml/min/1.73m^2^)	41.5	35.0	45.0	54.0	43.0	50.0	37.0	38.0	32.0	38.0	40.0
**Proteinuria** (mg/24h)	375	798	–	–	1517	5343	3395	4120	1076	940	510
**24h urine volume (ml/24h)**	2500	3000	–	–	–	1300	1750	1500	2200	2050	–
**ACR** (mg/g)	–	–	91	358	1334	6000	–	–	–	478	–
**Potassium** (mEq/l)	5.14	4.64	5.20	–	4.85	4.28	5.10	4.96	5.18	4.78	5.36
**HbA1c** (mmol/mol)	82	101	58	58	63	77	63	56	58	54	51
**Blood Pressure** (mmHg)	126/80	130/80	135/80	–	118/76	140/80	120/70	122/78	130/80	125/80	120/70

LRT, Leptin Replacement Therapy.

Given this excellent therapeutic response, in late 2021, when eGFR was 38 ml/min/1.73 m^2^, we tried again to withdraw losartan. At the following control visit, in 2022, kidney function and total proteinuria were both further improved: eGFR was 40 ml/min/1.73 m^2^ and proteinuria was 510 mg/24h. The patient showed good adherence to dietary restrictions and therapy. She is currently going on with a regular clinical follow-up, appearing in good clinical condition, no peripheral edema, no reported nausea, asthenia or fatigue.

## 3 Discussion

In the real life, the clinical scenario is often very different from what is shown in RCTs. This case report is a bright example, raising relevant points for discussion: *i*) double RAS blockade: pros and con; *ii*) antiproteinuric effect of SGLT2i; *iii*) leptin, metabolic control and proteinuria; *iv*) anaemia and competitive effects of SGLT2i and RAS blockers; *v*) status of the renal arteries.

ACEi and ARBs are well established drugs largely used for treating hypertension and reducing the progression of CKD in subjects with and without T2D (10), but their use in combination for treatment of proteinuria is not recommended by international guidelines (5). The ONTARGET and the ALTITUDE trials suggest that the combination of an ACEi and an ARB, despite reducing proteinuria more than a single agent, provides no benefit on major kidney outcomes and increases the incidence of adverse events, including hyperkalaemia, hypotension, and renal failure (11, 12). However, these trials enrolled aging patients with high cardiovascular risk and several comorbidities, naturally belonging to a high-risk group for cardiorenal events (13). On the other hand, these findings do not exclude the feasibility of this combined therapy in specific clinical conditions and specific disease stages. For example, in the presence of proteinuria, young individuals would gain more benefits and show fewer adverse reactions using for a limited time a dual RAS blockade strategy in the early stage of DKD. Moreover, double RAS blockade for treatment of nondiabetic nephropathy is more effective in reducing proteinuria and slowing the progression of nephropathy than single RAS blockade (14). Although international guidelines recommend a single drug treatment, using ACEi as first line option and reserving ARBs when ACEi are not tolerated or contraindicated (5), in special, rare patients, like the one we present here, combining a low dose of ACEIs and ARBs can be a feasible and successful option, especially in the attempt of reducing proteinuria, provided that close clinical monitoring can be guaranteed. Obviously, such approach should be reserved to a few, selected clinical cases, and cannot be applied on a large scale. We would also point out as, despite the main clinical benefits of RAS blockade are class effects, we should not assume that the results observed in this patient with ramipril+losartan can be obtained with any other ACEi+ARB combination.

In patients with CKD, albuminuria is a well-known determinant of further progression of kidney function decline (15). SGLT2i reduce albuminuria and slow eGFR loss independently of their glycaemic effects (1). Furthermore, as with RAS blockers, the extent of albuminuria reduction during the first months of treatment positively correlates with kidney and cardiovascular outcomes (16). While the positive effect of SGLT2i in patients with micro and macroalbuminuria have been largely established, the effect on nephrotic range proteinuria (NRP) is more controversial due to the different threshold used among studies and difficulty to include various etiologies of nephrotic syndrome other than DKD (17). In a recent secondary analysis of the EMPA-REG OUTCOME Trial (18) conducted in 112 patients with NRP at baseline (defined as UACR ≥2200 mg/g with any eGFR), a sustained UACR reduction ≥ 50% occurred in 58.8% of patients, and this percentage rise to more than 75% using a lower threshold of 30% UACR reduction. Similarly, a *post-hoc* analysis of the CREDENCE Trial (19) reported that patients whit UACR ≥ 3000 mg/g (n = 506) presented a lower relative reduction of albuminuria when compared to patients with UACR < 1000 mg/g and between 1000-3000 mg/g (14% *vs.* 31% and 29% respectively) but an absolute reduction of 341 mg/g. Although the effect on eGFR decline is consistent across all class of UACR, the results of DIAMOND Trial (58 subjects with non-diabetic CKD, *i.e.* eGFR ≥25 ml/min/1.73 m^2^ and UACR 500-3500 mg/g, treated with dapagliflozin for 6 weeks), showed a statistically significant reduction in proteinuria only in those with baseline eGFR >60 ml/min/1.73 m^2^, thus suggesting that proteinuria reduction may differ according to baseline eGFR (20).

Other factors may have contributed to slow the progression of kidney failure in this patient; among these, the improved metabolic control obtained with LRT. It should be also mentioned that LRT itself may reduce proteinuria in patients with lipodystrophy: in a recent open-label study, treatment with metreleptin for 24 months significantly reduced albuminuria and proteinuria in patients with GLD but not PLD (21). Of note, in that cohort, GLD patients presented baseline higher level of albuminuria and proteinuria than PLD patients. The authors reported baseline proteinuria as the only independent predictor for its further reduction with LRT, so the effect observed in GLD patients could probably depend on greater severity of kidney involvement (21). Since our patient presented extremely high proteinuria levels, we can speculate that LRT has played at least a concomitant role in the brilliant response we have observed.

Another aspect that should be considered, and frequently assessed when adding SGLT2i to a full-dose RAS blockade regimen, is red blood count and haemoglobin. Anaemia is largely prevalent in patients with T2D especially when CKD coexists (22). Many mechanisms are involved, but a reduced erythropoietin (EPO) production plays a central role. Low EPO levels can be due to tubulointerstitial damage, although they have been reported also in T2D subjects with preserved kidney function (23). Angiotensin-II, by inducing tubulointerstitial hypoxia, increases EPO secretion and up-regulates erythropoiesis. Treatment with RAS blockers has been associated to a reduction in haemoglobin levels, that could become relevant in subjects with CKD and/or T2D, needing more stimuli to support bone marrow function (24). In T2D, the avid glucose reabsorption by SGLTs increases glucose concentration in renal tubule-interstitium. Local glucotoxicity, together with release of pro-inflammatory molecules by renal epithelial cells, reduce EPO secretion (24). Increment of haemoglobin level and haematocrit have constantly been reported with SGLT2i (25). Despite a role of haemoconcentration due to volume contraction, the use of SGLT2i is associated with a transient increase of erythropoiesis, as reflected by an elevation of reticulocytes count that precedes the rise of haemoglobin and erythrocyte mass (24). Given their competing effect on erythropoiesis, the combined use of SGLT2i and RAS blockers should drive a close monitoring of blood count, as we did in our patient.

Lastly, the role of renal artery stenosis deserves consideration. Renovascular disease is a rare cause of CKD, whose prevalence increases in specific population of hypertensive patients and can be responsible of progressive end-stage renal disease. More often it is due to atherosclerotic changes in proximal renal artery (26). The kidney reacts to a contraction in arterial supply by reducing peripheral resistance so that, in the affected kidney, IRs appear lower than in the opposite side (“lateralization”) and rarely show the typical pattern of “tardus-parvus” with a slow acceleration and small systolic peak of the waveform. Other signs of chronic kidney hypoperfusion could then be found, like asymmetry in renal diameters and reduction of parenchymal thickness (27). Our patient, despite a relatively young age, has been exposed to a high, lifelong atherosclerotic burden due to metabolic derangement, hypercholesterolemia and hypertension that can explain the progressive development of multisite atherosclerotic vascular disease. Therefore, we regularly performed abdominal ultrasound follow-up with renal doppler examination. Although velocimetric values of right renal artery were suggestive of significant stenosis, IRs remain quite above 0.7 and both kidneys showed a specific progressive signs consistent with CKD of non-vascular origin (reducing of LL and loss of parenchymal differentiation).

## 4 Conclusion

In conclusion, SGLT2i are effective and safe in treating CKD and proteinuria of different aetiology. Their use in combination with RAS dual blocking, although requiring strict clinical monitoring for risk of hypotension and hyperkalaemia, can be considered a valid option in rare and complex disorders with massive protein loss and risk for a rapid progression to ESKD.

## Data availability statement

The original contributions presented in the study are included in the article/**Supplementary Material**. Further inquiries can be directed to the corresponding author.

## Ethics statement

Ethical approval was not provided for this study on human participants because single case report. Informed consent obtained. Written informed consent was obtained from the relevant individual for the publication of any potentially identifiable images or data included in this article.

## Author contributions

All Authors contributed to this work. EB: data acquisition, visualization, and writing – original draft. GC: data acquisition, visualization, and critical revision. SM: data acquisition and visualization. VO: evaluation of kidney biopsy. DG: data acquisition and execution of kidney biopsy. FS: data acquisition, visualization, and supervision. AS: data acquisition, visualization, supervision, and critical revision. All Authors approved the final version of the manuscript.
