# Supplementary material for: Canagliflozin on top of dual renin-angiotensin system blockade in a woman with partial acquired lipodystrophy, type 2 diabetes and severely proteinuric chronic kidney disease: a case report
Source: Front Endocrinol (Lausanne). 2023 May 19;14:1172468. doi: 10.3389/fendo.2023.1172468 (PMC10237351; doi:10.3389/fendo.2023.1172468)
Supplement: Supplementary file 1 [file Table_1.docx]

**Supplementary Table 1** Complete hormonal profile (2015), **A** and Autoimmunity screening (2015), **B**.

Normality ranges are reported within brackets.

**A B**

| **LH** (mUI/ml, 1.1-18.0) | 10.9 |
| --- | --- |
| **FSH** (mUI/ml, 1.3-18.0) | 3.8 |
| **Estradiol E2** (pg/ml, 40-340) | 181 |
| **Progesterone** (ng/ml, 0.1-1.5) | 0.70 |
| **17-OH Progesterone** (ng/ml, 0.6-2.3) | 1.83 |
| **Testosterone** (ng/ml, < 0.8) | 0.30 |
| **Androstenedione** (ng/ml, 0.32-0.42) | 3.51 |
| **DEHA-SO4** (µg/dl, 28-320) | 95 |
| **Cortisol** (µg/dl, 6.7-22.6) | 16.3 |
| **Prolactine** (ng/ml, 2.0-25.0) | 14.2 |
| **ACTH** (pg/ml, < 50) | 19 |
| **GH** (ng/ml, 0.01-3.60) | 1,09 |
| **PTH** (ng/ml, 8-40) | 29 |
| **TSH** (µU/ml, 0.400-4.000) | 0.853 |
| **fT3** (pg/ml, 2.70-5.70) | 3.49 |
| **fT4** (ng/dl, 0.70-1.70) | 0.92 |
| **Calcitonin** (pg/ml, < 11.5) | < 2.0 |

| **Rheumatoid factor** (U/ml, < 15.0) | 10.0 |
| --- | --- |
| **Complement C3c** (mg/dl, 90 – 180) | 131 |
| **Complement C4** (mg/dl, 10.0 – 40.0) | 26.9 |
| **Gastric Autoantibodies** (U/ml, < 10.0) | **46.5** |
| **Adrenal Autoantibodies** (U/ml, < 1.0) | 0.7 |
| **Antinuclear Antibodies** – **ANA** (neg) | 1:160 |
| **Extractable Nuclear Antigen Antibodies - ENA** (neg) | neg |
| **Anti-double Stranded DNA Antibodies** (neg) | neg |
| **Anti-Mitochondrial Antibodies** – **AMA** (neg) | neg |
| **Anti-Smooth Muscle Antibodies** – **ASMA** (neg) | neg |
| **Antineutrophil Cytoplasmic Antibodies** – **ANCA** (neg) | neg |
| **Anti-Scl-70 Antibodies** (neg) | neg |
| **Anticardiolipin Antibodies** – **ACLA IgG** (GPL-U/ml, < 10) | 1 |
| **Anticardiolipin Antibodies** – **ACLA IgM** (MPL-U/ml, < 10) | 2 |
| **Anti-Transglutaminase IgA** (U/ml, < 7.0) | 0.7 |
| **Anti-Transglutaminase IgG** (U/ml, < 7.0) | < 0.01 |
| **Glutamic Acid Decarboxylase Autoantibodies** (U/ml, < 1.0) | 0.8 |
